# Lower participation among immigrants in colorectal cancer screening in Norway

**DOI:** 10.3389/fpubh.2023.1254905

**Published:** 2023-09-26

**Authors:** Sameer Bhargava, Edoardo Botteri, Mona Berthelsen, Nadia Iqbal, Kristin Ranheim Randel, Øyvind Holme, Paula Berstad

**Affiliations:** ^1^Section for Colorectal Cancer Screening, Cancer Registry of Norway, Oslo, Norway; ^2^Department of Oncology, Akershus University Hospital, Lørenskog, Norway; ^3^Department of Research, Cancer Registry of Norway, Oslo, Norway; ^4^Department of Research, Sorlandet Hospital, Kristiansand, Norway; ^5^Institute of Health and Society, University of Oslo, Oslo, Norway

**Keywords:** immigrants, colorectal cancer, screening, minority health, access

## Abstract

**Introduction:**

Organized cancer screening programs should be equally accessible for all groups in society. We assessed differences in participation in colorectal cancer (CRC) screening among different immigrant groups.

**Methods:**

Between 2012 and 2019, 140,000 individuals aged 50 to 74 years were randomly invited to sigmoidoscopy or repeated faecal immunochemical test (FIT) in a CRC screening trial. In this study, we included 46,919 individuals invited to sigmoidoscopy and 70,018 invited to the first round of FIT between 2012 and 2017. We examined difference in participation between non-immigrants and immigrants, and within different immigrant groups by geographic area of origin, using logistic regression models, adjusted for several sociodemographic factors and health factors.

**Results:**

In total, we included 106,695 non-immigrants and 10,242 immigrants. The participation rate for FIT was 60% among non-immigrants, 58% among immigrants from Western countries and 37% among immigrants from non-Western countries. The participation rate for sigmoidoscopy was 53% among non-immigrants, 48% among immigrants from Western countries and 23% among immigrants from non-Western countries. Compared to non-immigrants, multivariate adjusted odds ratio for non-participation in FIT screening was 1.13 (95% confidence interval 1.04–1.23) and 1.82 (1.69–1.96) for immigrants from Western and non-Western countries. The corresponding numbers in sigmoidoscopy screening were 1.34 (1.21–1.48) and 2.83 (2.55–3.14). The lowest participation was observed in immigrants from Eastern Europe, Northern Africa and Western Asia, and South-Central Asia.

**Conclusion:**

Participation in CRC screening in Norway was particularly low among non-Western immigrants, which could put them at increased risk for late stage diagnosis of CRC. Participation was lower in sigmoidoscopy screening than in FIT screening, especially among immigrants from non-Western countries.

## 1. Introduction

Colorectal cancer (CRC) is one of the most common types of cancer, accounting for about 10% of cancer cases and 9% of cancer deaths in 2020 worldwide (1). The highest incidence of CRC has been observed in Europe, Northern America, Australia and New Zealand, while the lowest incidence has been observed in Africa and South-Central Asia. Norwegian women have the highest incidence of colon cancer among women in the world (1).

CRC screening with stool-based tests for occult blood and endoscopic methods have been shown to reduce CRC mortality (2). Given the high incidence of CRC, a population-based, nationwide CRC screening program administered by the Cancer Registry of Norway was launched in Norway in May 2022. Preceding the nationwide CRC screening program, a population-based pilot study in two counties in South East Norway compared screening with once-only sigmoidoscopy or four rounds of repeated faecal immunochemical test (FIT), showing participation rates of 52% for sigmoidoscopy, 58% for the first FIT round and 68% for three cumulative FIT rounds (3). The pilot study showed that both the participation rate and the detection rate for CRC was higher for repeated FIT than for sigmoidoscopy. The nationwide CRC screening program offers screening with FIT biennially for five rounds starting from the year participants turn 55. In the screening program, participants with a positive FIT receive an appointment for a follow-up colonoscopy.

In order for screening programs to be successful, people have to accept the offer of screening (4). In prior publications, we have shown that screening modality (sigmoidoscopy), male gender, younger age, low educational status, being unemployed, low income, being unmarried and prescription drug use were independent factors for non-participation (3, 5). Being an immigrant was another important factor associated with non-participation (5), but was not explored further in the prior publication. Lower CRC screening participation among immigrants compared to non-immigrants has also been observed in other countries (6–9).

Immigrants account for 16% of the population in Norway (10). While we have shown that being an immigrant was associated with non-participation in screening compared to non-immigrants, participation according to country of origin is unknown. This knowledge is important for screening organizers to be able to identify and address immigrant groups with low participation rates. While previous studies have shown lower CRC screening participation among immigrants compared to non-immigrants, this study adds to prior knowledge by exploring immigrants by screening modality and country of origin, allowing for identification of differences between non-immigrants and subgroups of immigrants that prior studies have not explored. In this study, we explore whether immigration had different impact on participation according to screening modality, country of origin and sociodemographic factors.

## 2. Materials and methods

This study is a sub-study of a population-based pilot study of the national CRC screening program, which enrolled individuals aged 50–74 years, residing in two geographical areas in South East Norway, randomly assigned in a 1:1 ratio to either once-only sigmoidoscopy or to FIT every second year for four rounds (3). For the current study, we included 116,938 individuals invited to sigmoidoscopy or the first round of FIT. While 140,000 individuals were invited to the pilot between 2012 and 2019, our population was somewhat less populous as data extraction was conducted in October 2017, before the end of the trial’s enrolment. The primary outcome of interest was CRC screening participation stratified by country of birth. One individual was excluded due to lack of information on country of birth, so the final population included 116,937 individuals.

### 2.1. Data sources and management

Data on marital status, country of birth, education, employment status and income were retrieved from Statistics Norway and referred to data from 2012. Data on drug prescriptions were obtained from the Norwegian Prescription Database. Data on screening participation was available from the project database for the CRC screening trial.

We defined all people born abroad with two foreign-born parents as immigrants. All other individuals were defined as non-immigrants. Country of origin was categorized into five levels, reflecting various bases of composition of country or area of origin. We report the categorization of immigrants in Supplementary Table S1.

### 2.2. Statistical analyzes

Odds ratios (OR) and 95% confidence intervals (CI) for non-participation were estimated using multivariate logistic regression models, in the entire population and separately in the two screening arms. OR and 95% CI for non-acceptance of the follow-up colonoscopy after a positive FIT result was estimated similarly. Non-acceptance of the follow-up colonoscopy after a positive sigmoidoscopy result was not estimated because of almost complete participation in that arm (97.8%). Based on previously published evidence (5, 11, 12), we adjusted the models for arm (unless stratified by arm), sex, age, education, employment status, household income, marital status, screening center, driving time to screening center, and use of antidiabetics, antipsychotics and anxiolytics. Age was categorized into: 50–55, 56–60, 61–65, 66–70, and > 70 years. Level of education was categorized into: primary school, high school, up to 4 years at university and more than 4 years at university. Employment was categorized into: employed, outside workforce/retired and unemployed. Household income comprised the sum of income from work, property income, taxable transfers, and tax-free transfers during one calendar year, and was divided into four categories based on quartiles. Marital status was dichotomized as: single/living alone and married/cohabiting. Driving distance comprised the driving distance in minutes by car from the home postal area to the screening center. We defined an individual as a user of antidiabetics, or user of antipsychotics and anxiolytics, if he/she received two prescriptions of that drug class during the year before invitation. In the analysis of heterogeneity, to test if the association between area of birth and participation was different between FIT and sigmoidoscopy, an arm*area of birth interaction term was added to the multivariable models. To test if the association between several factors (e.g., sex and education) and participation was different between individuals born in Norway and individuals born in other countries, the interaction terms (e.g., area of birth*sex and area of birth*education) were added to the multivariable model. The *p*-values associated with the interaction terms were then reported, and p-values <0.05 indicated a statistically significant heterogeneity between the groups of interest.

Statistical analyzes were conducted using SAS software, version 9.4 (SAS Institute, Inc., Cary, NC) and R, version 3.5.1.^1^

This study was approved by the Regional Committee for Medical Research Ethics in South East Norway (2011/1272).

## 3. Results

This study includes 116,937 participants invited for CRC screening (Table 1). Among the 70,018 participants offered FIT there were 63,843 non-immigrants and 6,175 immigrants. Participation rates for FIT were 59.7% for non-immigrants and 45.6% for immigrants. Among the 46,919 participants offered sigmoidoscopy there were 42,852 non-immigrants and 4,067 immigrants. Participation rates for sigmoidoscopy were 53.2% for non-immigrants and 33.2% for immigrants.

**Table 1:** Participation in colorectal cancer screening by area of birth.

	Country/area of birth	Both arms		FIT		Sigmoidoscopy	
		Invited No.	Attended No. (row %)	Invited No.	Attended No. (row %)	Invited No.	Attended No. (row %)
Level 0	All countries	116,937	65,089 (55.7)	70,018	40,930 (58.5)	46,919	24,159 (51.5)
Level 1	Norway	106,695	60,924 (57.1)	63,843	38,114 (59.7)	42,852	22,810 (53.2)
	Other countries	10,242	4,165 (40.7)	6,175	2,816 (45.6)	4,067	1,349 (33.2)
Level 2	Norway	106,695	60,924 (57.1)	63,843	38,114 (59.7)	42,852	22,810 (53.2)
	Western countries	4,221	2,258 (53.5)	2,532	1,456 (57.5)	1,689	802 (47.5)
	Non-Western countries	6,021	1,907 (31.7)	3,643	1,360 (37.3)	2,378	547 (23.0)
Level 3	Norway	106,695	60,924 (57.1)	63,843	38,114 (59.7)	42,852	22,810 (53.2)
	Western Europe	3,903	2,110 (54.1)	2,334	1,362 (58.4)	1,569	748 (47.7)
	Eastern Europe	2,677	793 (29.6)	1,619	575 (35.5)	1,058	218 (20.6)
	Northern America	295	138 (46.8)	188	88 (46.8)	107	50 (46.7)
	Latin America and the Caribbean	257	111 (43.2)	164	74 (45.1)	93	37 (39.8)
	Sub-Saharan Africa	388	115 (29.6)	227	84 (37.0)	161	31 (19.3)
	Northern Africa and Western Asia	669	186 (27.8)	410	121 (29.5)	259	65 (25.1)
	South-Central Asia	1,105	344 (31.1)	685	251 (36.6)	420	93 (22.1)
	Eastern Asia and the Pacific	925	358 (38.7)	538	255 (47.4)	387	103 (26.6)
	Australia/New Zealand	23	10 (43.5)	10	6 (60.0)	13	4 (30.8)
Level 4	Norway	106,695	60,924 (57.1)	63,843	38,114 (59.7)	42,852	22,810 (53.2)
	The other Nordic Countries	2,332	1,277 (54.8)	1,418	838 (59.1)	914	439 (48.0)
	Rest of Western Europe	1,571	833 (53.0)	916	524 (57.2)	655	309 (47.2)
	Eastern Europe	2,677	793 (29.6)	1,619	575 (35.5)	1,058	218 (20.6)
	Northern America	295	138 (46.8)	188	88 (46.8)	107	50 (46.7)
	Caribbean and Central America	46	24 (52.2)	25	13 (52.0)	21	11 (52.4)
	South America	211	87 (41.2)	139	61 (43.9)	72	26 (36.1)
	Middle and Western Africa	69	26 (37.7)	44	21 (47.7)	25	5 (20.0)
	Eastern Africa	304	79 (26.0)	172	54 (31.4)	132	25 (18.9)
	Southern Africa	15	10 (66.7)	11	9 (81.8)	<5	<5
	Northern Africa	428	113 (26.4)	258	69 (26.7)	170	44 (25.9)
	Western Asia	241	73 (30.3)	152	52 (34.2)	89	21 (23.6)
	South-Central Asia	1,105	344 (31.1)	685	251 (36.6)	420	93 (22.1)
	Southeast Asia and the Pacific	730	268 (36.7)	420	189 (45.0)	310	79 (25.5)
	Eastern Asia	195	90 (46.2)	118	66 (55.9)	77	24 (31.2)
	Australia/New Zealand	23	10 (43.5)	10	6 (60.0)	13	4 (30.8)

The levels refer to countries/areas categorized in the following way: Level 0 describes all participants. Level 1 describes participants born in Norway and all other countries combined. Level 2 describes participants born in Norway and major geographical areas. Level 4 describes participants born in Norway and minor geographical areas.

Among immigrants, participation rates for FIT were 57.5% for immigrants from Western countries and 37.3% for immigrants from non-Western countries (Table 1), The lowest participation rates for FIT were observed among immigrants from the regions of Northern Africa (26.7%), Eastern Africa (31.4%), Western Asia (34.2%) and Eastern Europe (35.5%), and from the countries of Somalia (18.2%), Kosovo (24.0%), Iraq (27.7%) and Pakistan (27.8%) (Tables 1 2). Participation rates for sigmoidoscopy were 47.5% for immigrants from Western countries and 23.0% for immigrants from non-Western countries (Table 1). The lowest participation rates for sigmoidoscopy were observed among immigrants from the regions of Eastern Africa (18.9%), Middle and Western Africa (20.0%), Eastern Europe (20.6%) and South-Central Asia (22.1%), and from the countries of Somalia (8.9%), Kosovo (14.9%), India (14.3%), Pakistan (16.1%) and Poland (18.3%).

**Table 2:** Participation in colorectal cancer screening by country of birth in descending order of participation for both arms combined.

	Country of birth	Both arms		FIT		Sigmoidoscopy	
		Invited No.	Attended No. (row %)	Invited No.	Attended No. (row %)	Invited No.	Attended No. (row %)
Level 5	Norway	106,695	60,924 (57.1)	63,843	38,114 (59.7)	42,852	22,810 (53.2)
	Denmark	857	484 (56.5)	519	310 (59.7)	338	174 (51.5)
	Netherlands	178	100 (56.2)	104	62 (59.6)	74	38 (51.4)
	United Kingdom	540	300 (55.6)	324	190 (58.6)	216	110 (50.9)
	Finland	194	107 (55.2)	127	77 (60.6)	67	30 (44.8)
	Iceland	145	80 (55.2)	90	53 (58.9)	55	27 (49.1)
	Sweden	1,117	597 (53.4)	668	390 (58.4)	449	207 (46.1)
	Germany	508	252 (49.6)	269	145 (53.9)	239	107 (44.8)
	United States of America	261	123 (47.1)	169	80 (47.3)	92	43 (46.7)
	China	116	50 (43.1)	70	39 (55.7)	46	11 (23.9)
	Chile	124	52 (41.9)	85	38 (44.7)	39	14 (35.9)
	Iran	428	168 (39.3)	257	118 (45.9)	171	50 (29.2)
	Vietnam	362	136 (37.6)	201	98 (48.8)	161	38 (23.6)
	Thailand	104	38 (36.5)	71	30 (42.3)	33	8 (24.2)
	Philippines	189	63 (33.3)	110	40 (36.4)	79	23 (29.1)
	Russia	171	55 (32.2)	107	38 (35.5)	64	17 (26.6)
	Turkey	135	43 (31.9)	92	31 (33.7)	43	12 (27.9)
	Poland	1,002	293 (29.2)	613	222 (36.2)	389	71 (18.3)
	Bosnia and Herzegovina	560	161 (28.8)	322	115 (35.7)	238	46 (19.3)
	India	186	51 (27.4)	123	42 (34.1)	63	9 (14.3)
	Iraq	321	83 (25.9)	191	53 (27.7)	130	30 (23.1)
	Pakistan	306	72 (23.5)	194	54 (27.8)	112	18 (16.1)
	Kosovo	268	54 (20.1)	154	37 (24.0)	114	17 (14.9)
	Somalia	144	21 (14.6)	88	16 (18.2)	56	5 (8.9)

Only countries with at least 100 invitees were selected. Level 5 describes individual countries of birth.

**Figure 1:**
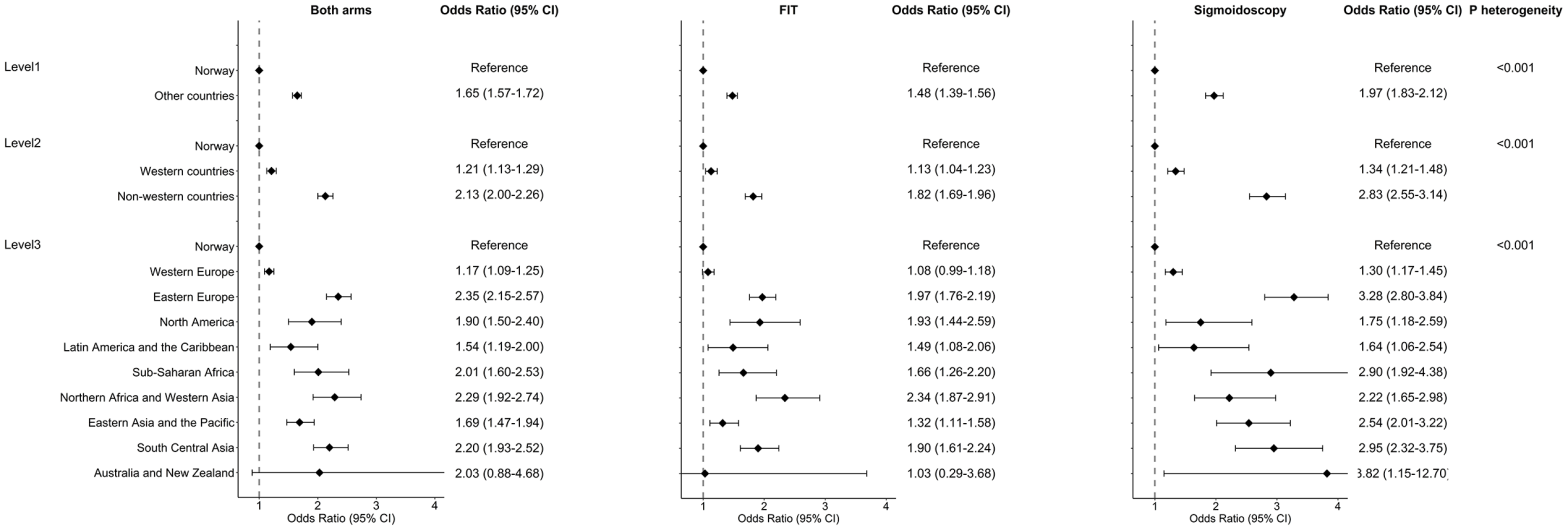
Odds ratios for non-participation in colorectal cancer screening, comparing individuals born in Norway and individuals born in other countries. Odds ratios for non-participation with 95% confidence interval (CI) deriving from multivariable logistic regressions, adjusted for arm (unless stratified by arm), sex, age, education, occupation, household, marital status, screening center, driving time to screening center, and use of antidiabetics, antipsychotics and anxiolytics. *p* for heterogeneity was calculated to evaluate differences in ORs between arms. Graphical CI were truncated for New Zealand and Australia.

Non-participation in screening remained higher for immigrants versus non-immigrants after adjusting for sociodemographic and health factors (Figure 1). In both arms, immigrants from non-Western countries had a lower participation compared to immigrants from Western countries. For FIT, OR_adj_ for non-participation among immigrants versus non-immigrants was 1.48 (CI 1.39–1.56). Immigrants from Western Europe had a similar participation compared to non-immigrants in FIT (OR_adj_ 1.08, CI 0.99–1.18). For all other regions, immigrants had higher OR_adj_ for non-participation in FIT (except for Australia/New Zealand possibly due to low numbers). For sigmoidoscopy, OR_adj_ for non-participation among immigrants versus non-immigrants was 1.97 (CI 1.83–2.12). For all regions, immigrants had higher OR_adj_ for non-participation in sigmoidoscopy. P for heterogeneity between FIT and sigmoidoscopy screening was <0.001 for all analysis in Figure 1. Among immigrants invited to colonoscopy after a positive FIT, OR_adj_ for non-participation were 1.10 (CI 0.49–2.46) among immigrants from Western countries and 3.37 (CI 1.91–5.97) among immigrants from non-Western countries (Table 3).

**Table 3:** Individuals invited to colonoscopy after a positive fæcal immunochemical test and multivariable logistic regression analyzes with odds ratios for non-acceptance of colonoscopy.

	Country/area of birth	Invited to colonoscopy	Accepted colonoscopy (row %)	OR (95% CI) for non-participation
Level 1	Norway	3,069	2,895 (94.3)	Reference
	Other countries	231	205 (88.7)	2.13 (1.34–3.39)
Level 2	Norway	3,069	2,895 (94.3)	Reference
	Western countries	117	110 (94.0)	1.10 (0.49–2.46)
	Non-Western countries	114	95 (83.3)	3.37 (1.91–5.97)

Odds ratios (ORs) for non-acceptance of colonoscopy with 95% confidence interval (CI) deriving from multivariable logistic regressions, adjusted for sex, age, education, occupation, household, marital status, screening center, driving time to screening center, and use of antidiabetics, antipsychotics and anxiolytics.

The same sociodemographic factors were associated with lower participation in screening both for immigrants and for non-immigrants, namely being randomized into the sigmoidoscopy arm, young age, no higher education, being unemployed, being single/widowed and use of antipsychotic and/or anxiolytic drugs (Table 4). Educational status, employment status, marital status and use of antipsychotic and anxiolytic drugs had a stronger association with participation among non-immigrants, while screening arm and age had a stronger association with participation among immigrants. Screening arm was particularly associated with participation in non-Western immigrants (Supplementary Table S2).

**Table 4:** Multivariate odds ratios for non-participation in colorectal cancer screening according to sociodemographic characteristics, in individuals born in Norway and individuals born in other countries.

	Strata	Born in Norway			Born in other countries			*p* heterogeneity
		Invited No.	Attended No. (row %)	OR (95% CI)	Invited No.	Attended No. (row %)	OR (95% CI)	
Arm	Sigmoidoscopy	42,852	22,810 (53.2)	1.33 (1.30–1.37)	4,067	1,349 (33.2)	1.78 (1.63–1.94)	<0.001
	FIT	63,843	38,114 (59.7)	Reference	6,175	2,816 (45.6)	Reference	
Sex	Males	52,397	29,134 (55.6)	1.28 (1.25–1.32)	5,241	1,931 (36.8)	1.39 (1.27–1.51)	0.090
	Females	54,298	31,790 (58.5)	Reference	5,001	2,234 (44.7)	Reference	
Age (years)	50–55	19,386	10,199 (52.6)	1.64 (1.56–1.72)	2,821	988 (35.0)	2.29 (1.96–2.66)	<0.001
	56–60	24,443	13,554 (55.5)	1.41 (1.35–1.48)	2,766	1,076 (38.9)	1.85 (1.59–2.15)	
	61–65	22,502	13,378 (59.5)	1.13 (1.08–1.18)	1,844	777 (42.1)	1.57 (1.34–1.84)	
	66–70	22,325	13,788 (61.8)	0.89 (0.85–0.93)	1,553	732 (47.1)	1.14 (0.98–1.34)	
	>70	18,039	10,005 (55.5)	Reference	1,258	592 (47.1)	Reference	
Education	Primary school	23,047	9,933 (43.1)	1.93 (1.83–2.05)	2,493	813 (32.6)	1.54 (1.32–1.80)	<0.001
	High school	50,389	29,139 (57.8)	1.26 (1.20–1.33)	3,114	1,327 (42.6)	1.11 (0.96–1.28)	
	1–4 years university	23,541	15,544 (66.0)	0.98 (0.93–1.04)	2,564	1,287 (50.2)	0.95 (0.82–1.10)	
	>4 years university	9,163	6,126 (66.9)	Reference	1,174	588 (50.1)	Reference	
Occupation	Retired	41,386	21,542 (52.1)	1.20 (1.16–1.24)	4,567	1,616 (35.4)	1.40 (1.26–1.55)	0.037
	Unemployed	567	251 (44.3)	1.53 (1.28–1.82)	206	47 (22.8)	1.91 (1.36–2.70)	
	Employed	64,704	39,126 (60.5)	Reference	5,459	2,501 (45.8)	Reference	
Household income (NOK)	≤484,000	25,500	10,715 (42.0)	1.75 (1.66–1.84)	3,715	1,090 (29.3)	1.79 (1.56–2.06)	0.798
	484,001–755,000	26,776	15,393 (57.5)	1.21 (1.16–1.26)	2,435	1,036 (42.5)	1.23 (1.08–1.40)	
	755,001–1,130,000	27,083	16,791 (62.0)	1.09 (1.05–1.13)	2,157	1,038 (48.1)	1.03 (0.91–1.18)	
	>1,130,000	27,280	18,019 (66.1)	Reference	1,923	1,000 (52.0)	Reference	
Marital status	Single/widow	27,234	12,219 (44.9)	1.39 (1.34–1.44)	2,359	824 (34.9)	1.15 (1.03–1.29)	0.006
	Cohabit/married	79,452	48,702 (61.3)	Reference	7,851	3,336 (42.5)	Reference	
Driving distance (minutes)	> 40	21,078	11,132 (52.8)	1.11 (1.07–1.14)	1,368	507 (37.1)	1.17 (1.05–1.30)	0.585
	21–40	39,610	22,101 (55.8)	1.19 (1.15–1.23)	3,364	1,299 (38.6)	1.29 (1.13–1.48)	
	≤ 20	44,285	27,064 (61.1)	Reference	4,994	2,252 (45.1)	Reference	
Use of antidiabetics	Yes	6,847	3,168 (46.3)	1.38 (1.31–1.45)	1,025	333 (32.5)	1.30 (1.12–1.50)	0.450
	No	99,848	57,756 (57.8)	Reference	9,217	3,832 (41.6)	Reference	
Use of antipsychotics and/or anxiolytics	Yes	8,740	3,461 (39.6)	1.62 (1.54–1.70)	569	187 (32.9)	1.21 (1.00–1.46)	0.003
	No	97,955	57,463 (58.7)	Reference	9,673	3,978 (41.1)	Reference	

Odds ratios (ORs) for non-participation with 95% confidence interval (CI) deriving from multivariable logistic regressions. *p*-value for heterogeneity was calculated to evaluate differences in ORs between individuals born in Norway and individuals born in other countries.

OR_adj_ for non-participation in sigmoidoscopy versus FIT was 1.33 (CI 1.30–1.37) for non-immigrants, 1.57 (CI 1.38–1.78) for immigrants from Western countries and 2.05 (CI 1.82–2.31) for immigrants from non-Western countries (Supplementary Table S2). OR_adj_ for non-participation for primary school as highest level of education versus >4 years of university was 1.93 (CI 1.83–2.05) for non-immigrants, 1.47 (CI 1.16–1.86) for immigrants from Western countries and 1.42 (CI 1.14–1.77) for immigrants from non-Western countries. Use of antidiabetic and antipsychotic and/or anxiolytic drugs was associated with non-participation for non-immigrants and immigrants from Western countries, but not for immigrants from non-Western countries.

## 4. Discussion

In this study, we showed that immigrants participated less often than non-immigrants in the CRC screening pilot study in Norway in the period 2012–2017. Immigrants from non-Western countries had particularly low participation rates, especially in sigmoidoscopy screening. Participation rates remained lower after adjusting for sociodemographic factors.

In Denmark, participation rates in screening with immunochemical faecal occult blood test (FOBT) in 2014 and 2015 were 65.9% among immigrants, 61.5% among immigrants from Western countries and 53.0% among immigrants from non-Western countries (8). In Finland, immigrants were found to have lower participation rates in screening with guaiac FOBT compared to non-immigrants between 2004 and 2016 (9). The lowest participation rates among men were observed for immigrants from non-European countries (44.1% versus 61.4% for non-immigrant men), while the lowest participation rates among women were observed for immigrants from Europe outside the Nordic countries (55.0% versus 75.6% among non-immigrant women). An Italian survey-based study with data from 2013 showed that immigrants had lower participation rates in screening with FIT compared to non-immigrants (34.3% versus 51.3%), and that women participated more often than men for all age groups both among immigrants and non-immigrants (7). The Italian study showed similar participation in follow-up colonoscopy for immigrants versus non-immigrants [80.5% versus 81.8%, relative rate (95% CI) 0.98 (0.94–1.01)]. A survey-based study from Canada using data from 2005 to 2012 reported that CRC screening adherence was 53.9% among non-immigrants, 50.3% among non-recent immigrants and only 22.5% among recent immigrants (13). Our study uses high-quality registries to build on these findings and adds granularity by stratifying immigrants according to country of birth and screening method.

The United Nations Sustainable Development Goal (SDG) 3 aims to “ensure healthy lives and promote well-being for all at all ages” (14). A target of SDG 3 is to achieve universal health coverage with access to health care services. Immigrants have not only been found to have lower participation in CRC screening, but also in breast and cervical cancer screening (15, 16). In order to achieve the targets set in SDG 3, policy makers and service providers should strive to increase immigrants’ participation in organized cancer screening. Several barriers to cancer screening have been identified among immigrants, including language barriers, challenges navigating health care systems, low health literacy levels/lack of awareness of disorder and cultural beliefs (17, 18). A study from Flanders in Belgium showed that people with Belgian or Dutch nationality had higher FIT screening participation rates compared to people with all other nationalities (Dutch nationals do not experience a language barrier in Flanders) (19). The study observed that having a Belgian or Dutch current nationality was associated with higher screening participation regardless of nationality at birth. The authors suggest that having a Belgian nationality is likely to be a proxy for year of immigration, and that people who have gained a Belgian nationality are more likely to be well integrated in society. Service providers should target known barriers in order to increase participation in CRC screening for subgroups at risk for non-participation. Such strategies could include considering how personal factors (for instance language barriers) may interact with systemic barriers (for instance organization of health care services) and prevent accessibility for participants (20).

Differences in participation between immigrants and non-immigrants observed in our study were more pronounced for sigmoidoscopy, which is a more invasive screening method, compared to FIT. Screening programs require high participation rates in order to perform adequately. If the low participation among immigrants for sigmoidoscopy is due to its invasive nature, it is possible that FIT is more optimal for immigrants than endoscopic screening methods, in order to achieve as high participation rates as possible. However, a positive FIT requires follow-up for diagnosis and subsequent treatment. The low acceptance for both screening with sigmoidoscopy and follow-up colonoscopy after FIT among immigrants from non-Western countries is a major concern that can potentially impair mortality reduction in these groups.

The incidence of CRC has been shown to be higher among non-immigrants than immigrants, with immigrants from non-Western countries having the lowest rates of CRC (21). One could thus ask whether immigrants from non-Western countries are less concerned about CRC or have less awareness of the disease, as it is less common both in their countries of birth and among their diaspora abroad. However, it is important with early diagnosis even for low-risk populations such as immigrants from Eastern Asia and Sub-Saharan Africa. Immigrants from these areas have had a tendency towards more advanced disease (22). Further, it is important that non-participation in CRC screening among immigrants is not replicated by their children, as the incidence of CRC may be higher among children of immigrants to countries with high incidence of CRC compared to both their parents and the host population (23).

The 144 immigrants from Somalia in the study population had the lowest participation in screening, regardless of screening modality. It is of particular concern that less than 10% of immigrants from Somalia attended sigmoidoscopy. The approximately 28,000 immigrants from Somalia in Norway make up the sixth most populous immigrant group in Norway (10). If descendants of immigrants are included, people with a background from Somalia make up the third most populous group with a foreign background, of whom a majority are yet to enter the age group targeted by the CRC screening program. Immigrants from Somalia have by far the lowest participation in breast cancer screening, and also low participation in cervical cancer screening (15, 24), and may be at increased risk for morbidity and mortality from multiple cancer types. While a qualitative study explored individual, sociocultural and system-related factors influencing cervical cancer screening participation in Norway among Pakistani and Somali immigrant women combined (25), further qualitative studies that focus on cancer screening participation specifically among Somali immigrants across different screening programs are required.

We also observed low participation in CRC screening among immigrants from Poland, especially for sigmoidoscopy. Polish immigrants are the most populous immigrant group in Norway including more than 107,000 individuals (10). We recently performed a qualitative study exploring Polish immigrants’ access to CRC screening in Norway (26). The immigrants from Poland in this study had trust in CRC screening, but often did not understand that FIT was a method of CRC screening. Further, some of the participants had either attended colonoscopy as a screening examination in Poland or knew of people who had undergone colonoscopy in Poland. Low CRC screening rates among Polish immigrants could thus indicate that they go for screening in Poland rather than in Norway. However, a recent trial showed clearly lower participation in population-based colonoscopy screening in Poland than in Norway (33 vs. 61%, respectively), indicating a lower acceptance for this examination in the Polish population (27). Unless the colonoscopy numbers from Poland are low due to a high degree of opportunistic, private screening in Poland, it could appear that Polish people remain underscreened. While one may discuss whether screening in their country of birth could be part of the explanation for low participation for immigrants from countries geographically close to Norway, it is less likely to be the case for immigrants from more distant countries.

There were substantial differences in screening participation between immigrants from different parts of the world. While immigrants from most regions had higher participation in FIT compared to sigmoidoscopy, there was no difference between participation in FIT (46.8%) and sigmoidoscopy (46.7%) among immigrants from Northern America. A higher acceptance for the more invasive procedure among immigrants from Northern America could reflect the higher use of colonoscopy compared to stool-based screening tests in the United States (28). A similar lack of difference between participation in FIT and sigmoidoscopy was observed among immigrants from Northern Africa, but with lower participation rates in both arms; 26.7% participation in FIT and 25.9% participation in sigmoidoscopy.

We observed that non-participation decreased with increasing age both for non-immigrants and immigrants, and the effect appeared to be greatest among immigrants from Western countries. This could reflect elements of care ethics (29), as people in their early 50’s are more likely to have competing interest related to following up their children and older adults parents compared to people in their 70’s. This may be truer for immigrants than for non-immigrants reflecting the greater effect observed among immigrants, as most immigrant groups are considered to be more family oriented compared to the majority population in Norway. Further, older immigrants may have grown-up children who can help them navigate the healthcare system in Norway.

Being invited to sigmoidoscopy compared to FIT screening and being retired appeared to be more strongly associated with non-participation particularly among immigrants from non-Western countries compared to non-immigrants. In contrast, the association between low education level and use of diabetic or psychotropic medication, and non-participation in CRC screening shown in non-immigrants was less pronounced in non-Western immigrants. Overall, our results suggest that the sociodemographic inequalities in participation slightly differed between non-immigrants and immigrant groups, and that the largest predictor for non-participation for non-Western immigrants was invitation to sigmoidoscopy compared to FIT screening.

A target of SDG 3 is to reduce premature mortality from non-communicable disease through prevention and treatment, and mortality rate attributed to cancer is an indicator to measure this target (14). The aim of cancer screening programs is to reduce mortality by early detection of disease. The low CRC screening participation rates among immigrants observed in several countries are of particular concern if it results in increased mortality from the disease. Some studies have failed to identify disadvantages in survival among immigrants with CRC (30, 31). Service providers should, however, continue to strive to improve participation among immigrants. Survival analyzes may be limited by several factors that can overestimate survival or prevent statistically significant findings, including a low number of cancer cases, especially in subgroups, and a risk that people may travel to their country of origin when they receive a cancer diagnosis. Further, immigrants may have a higher risk of CRC than people in their country of birth, and children of immigrants may have higher risk of CRC than their parents, indicating risk adaptation (23, 32, 33). Finally, if there is no difference in survival between immigrants and non-immigrants despite lower CRC screening participation among immigrants, it is possible that immigrants might even have had preferential survival with similar participation as non-immigrants.

Major strengths of the present study are the study design with a randomized controlled trial, large sample size and data on immigration status, country of birth, sociodemographic status and drug use drawn from population registries, ensuring low number of missing values and low selection bias. The study comprises complete information about screening participation for all invitees. It is, however, a limitation that the study population is geographically restricted to an area surrounding, but not including, the capitol city, Oslo, which has the most populous immigrant population in Norway. Another limitation is that we categorized immigrants only according to their country of birth. While this categorization could be considered a surrogate for societal and cultural factors, it is a poor marker for other factors that may be of relevance, including reasons for migration, post-migration stress and experience with public services in the migrants’ country of origin. The youngest participants in our study were born in the early 1960’s. Net migration to Norway was not positive before the 1970’s, meaning that we were unable to explore attendance among descendants of immigrants. Further, transnationalism explains an increasing, multilateral and dynamic exchange of people, thoughts and practices between countries, which we are not able to pick up through a registry-based study (34). Finally, participants who appear unscreened in our study might have undergone CRC screening outside the study, for instance in their country of birth.

## 5. Conclusion

Immigrants had lower participation rates in CRC screening in Norway compared to non-immigrants. The lower participation was observed across immigrant groups and regardless of screening method. This potentially puts immigrants at risk for increased morbidity and mortality from CRC. Immigrants from non-western countries were at particular risk for non-participation in screening, especially for sigmoidoscopy screening. Results from the present study indicate that, for the vulnerable immigrant groups, FIT is related to a higher access to CRC screening than sigmoidoscopy, but the acceptance for invasive follow-up examination with colonoscopy is low. Our results should be of interest to health professionals and service providers working with preventive health or migrant health in other high-income countries.

## Data availability statement

The datasets presented in this article are not readily available because access to research data for external investigators will require approval from the Norwegian Regional Committee for Medical and Health Research Ethics and the Bowel cancer screening in Norway steering committee (information available on the project website: https://www.kreftregisteret.no/screening/Tarmscreeningpiloten/). Research data are not openly available because of the principles and conditions set out in articles 6 [1] (e) and 9 [2] (j) of the General Data Protection Regulation (GDPR). Requests to access the dataset should be directed to the corresponding author. Requests to access the datasets should be directed to https://www.kreftregisteret.no/screening/Tarmscreeningpiloten/.

## Author contributions

SB: Conceptualization, Data curation, Funding acquisition, Investigation, Methodology, Project administration, Resources, Validation, Visualization, Writing – original draft, Writing – review & editing. EB: Conceptualization, Data curation, Formal analysis, Investigation, Methodology, Resources, Supervision, Validation, Visualization, Writing – original draft, Writing – review & editing. MB: Conceptualization, Data curation, Investigation, Methodology, Resources, Validation, Visualization, Writing – original draft, Writing – review & editing. NI: Conceptualization, Resources, Validation, Visualization, Writing – review & editing. KR: Conceptualization, Funding acquisition, Resources, Supervision, Validation, Visualization, Writing – review & editing. ØH: Conceptualization: Funding acquisition, Resources, Supervision, Validation, Visualization, Writing – review & editing. PB: Conceptualization, Data curation, Funding acquisition, Investigation, Methodology, Project administration, Resources, Supervision, Validation, Visualization, Writing – original draft, Writing – review & editing.

## Funding

SB and NI are supported by a research grant from the Norwegian Cancer Society, for which PB was the applicant and project leader (213396-2019). The trial was funded by the Norwegian Parliament through the national budget in preparation for the nationwide colorectal cancer screening program that started in 2022. The bowel preparation used for colonoscopy was provided free of charge by Ferring Pharmaceuticals. The funders of the study were not involved in conducting the study.

## Conflict of interest

SB is due to receive personal fees from Gilead outside the submitted work.

The remaining authors declare that the research was conducted in the absence of any commercial or financial relationships that could be construed as a potential conflict of interest.

## Publisher’s note

All claims expressed in this article are solely those of the authors and do not necessarily represent those of their affiliated organizations, or those of the publisher, the editors and the reviewers. Any product that may be evaluated in this article, or claim that may be made by its manufacturer, is not guaranteed or endorsed by the publisher.

## Footnotes

^1^http://cra.r-project.org

## Abbreviations

CI: Confidence Interval
CRC: Colorectal Cancer
FIT: Faecal Immunochemical Test
FOBT: Faecal Occult Blood Test
OR: Odds Ratio
SDG: Sustainable Development Goal
