# Supplementary material for: Lower participation among immigrants in colorectal cancer screening in Norway
Source: Front Public Health. 2023 Sep 26;11:1254905. doi: 10.3389/fpubh.2023.1254905 (PMC10562536; doi:10.3389/fpubh.2023.1254905)
Supplement: Supplementary file 1 [file Table_2.docx]

Supplementary Material

Article Title

Sameer Bhargava*, Edoardo Botteri, Mona Berthelsen, Nadia Iqbal, Kristin Ranheim Randel, Øyvind Holme, Paula Berstad

*** Correspondence:** Sameer Bhargava: [Sameer.bhargava@kreftregisteret.no](mailto:Sameer.bhargava@kreftregisteret.no)

**Supplementary Table 1.** Categorisation of participants into groups for comparison according to country of birth.

| Level 1 | Level 2 | Level 3 | Level 4 | Level 5 |
| --- | --- | --- | --- | --- |
| Norway | Norway | Norway | Norway | Norway |
| Immigrants | Western countries | Western Europe | The other Nordic countries | Denmark, Greenland, Finland, Faroe Islands, Iceland and Sweden |
|  |  |  | Rest of Western Europe | Ireland, United Kingdom, Belgium, France, the Netherlands, Liechtenstein, Luxembourg, Monaco, Switzerland, Germany, Austria, Andorra, Italy, Malta, Portugal, San Marino, and Spain |
|  |  | Northern America | Northern America | Canada and the United States of America |
|  |  | Australia/New Zealand | Australia/New Zealand | Australia and New Zealand |
|  | Non-Western countries | Eastern Europe | Eastern Europe | Estonia, Latvia, Lithuania, Albania, Croatia, Cyprus, Greece, Slovenia, Bosnia-Herzegovina, Macedonia, Serbia, Montenegro, Kosovo, Bulgaria, Belarus, Poland, Romania, Moldova, Russia, Ukraine, Hungary, Slovakia and the Czech Republic |
|  |  | Latin America and the Caribbean | Caribbean and Central America | United States Virgin Islands, Barbados, Bahamas, Cayman Islands, Cuba, Dominica, Dominican Republic, Grenada, Guadeloupe, Haiti, Jamaica, Aruba, Curacao, Saint Lucia, Trinidad and Tobago, Puerto Rico, Belize, Costa Rica, Guatemala, Honduras, Mexico, Nicaragua, Panama and El Salvador |
|  |  |  | South America | Argentina, Bolivia, Brazil, Guyana, Chile, Colombia, Ecuador, Paraguay, Peru, Suriname, Uruguay and Venezuela |
|  |  | Sub-Saharan Africa | Middle and Western Africa | Angola, Cameroon, Congo-Brazzaville, Congo, Sao Tome and Principe, Central African Republic, Benin, Ivory Coast, Gambia, Ghana, Guinea, Guinea-Bissau, Cape Verde, Liberia, Nigeria, Senegal, Sierra Leone, Togo and Burkina Faso |
|  |  |  | Eastern Africa | Burundi, Comoros, Eritrea, Ethiopia, Djibouti, Kenya, Madagascar, Malawi, Mauritius, Mozambique, Réunion, Zimbabwe, Rwanda, Seychelles, Somalia, South Sudan, Tanzania, Uganda and Zambia |
|  |  |  | Southern Africa | Botswana, Lesotho, Namibia, Swaziland and South Africa |
|  |  | Northern Africa and Western Asia (Middle East) | Northern Africa | Algeria, Egypt, Libya, Morocco, Western Sahara, Sudan and Tunisia |
|  |  |  | Western Asia | Turkey, Armenia, Azerbaijan, United Arab Emirates, Georgia, Iraq, Israel, Jordan, Kuwait, Lebanon, Palestine, Saudi Arabia, Syria and Yemen |
|  |  | South-Central Asia | South-Central Asia | Afghanistan, Bangladesh, Bhutan, Sri Lanka, India, Iran, Kazakhstan, Kyrgyzstan, Maldives, Nepal, Pakistan, Tajikistan, Turkmenistan and Uzbekistan |
|  |  | Eastern Asia and the Pacific | Southeast Asia and the Pacific | Brunei, Myanmar, the Philippines, Indonesia, Cambodia, Laos, Malaysia, Timor-Leste, Singapore, Thailand, Vietnam and Papua New Guinea |
|  |  |  | Eastern Asia | Taiwan, Hong Kong, Japan, China, North Korea, South Korea, Macao and Mongolia |

**Supplementary Table 2.** Multivariate odds ratios for non-participation in colorectal cancer screening according to sociodemographic characteristics, in individuals born in Norway, individuals born in Western countries and individuals born in non-Western countries.

|  |  | **Born in Norway** | | | **Born in Western countries** | | | **Born in non-Western countries** | | | **P _heterogeneity_** |
| --- | --- | --- | --- | --- | --- | --- | --- | --- | --- | --- | --- |
|  | **Strata** | **Invited**  **No.** | **Attended**  **No. (row %)** | **OR (95% CI)** | **Invited**  **No.** | **Attended**  **No. (row %)** | **OR (95% CI)** | **Invited**  **No.** | **Attended**  **No. (row %)** | **OR (95% CI)** |  |
| **Arm** | **Sigmoidoscopy** | 42,852 | 22,810 (53.2) | 1.33 (1.30-1.37) | 1,689 | 802 (47.5) | 1.57 (1.38-1.78) | 2,378 | 547 (23.0) | 2.05 (1.82-2.31) | <0.001 |
|  | **FIT** | 63,843 | 38,114 (59.7) | Reference | 2,532 | 1,456 (57.5) | Reference | 3,643 | 1,360 (37.3) | Reference |  |
| **Sex** | **Males** | 52,397 | 29,134 (55.6) | 1.28 (1.25-1.32) | 2,033 | 968 (47.6) | 1.55 (1.36-1.77) | 3,208 | 963 (30.0) | 1.24 (1.10-1.39) | 0.049 |
|  | **Females** | 54,298 | 31,790 (58.5) | Reference | 2,188 | 1,290 (59.0) | Reference | 2,813 | 944 (33.6) | Reference |  |
| **Age (years)** | **50-55** | 19,386 | 10,199 (52.6) | 1.64 (1.56-1.72) | 837 | 403 (48.1) | 2.06 (1.63-2.61) | 1,984 | 585 (29.5) | 1.40 (1.10-1.78) | 0.042 |
|  | **56-60** | 24,443 | 13,554 (55.5) | 1.41 (1.35-1.48) | 940 | 470 (50.0) | 1.83 (1.46-2.30) | 1,826 | 606 (33.2) | 1.12 (0.88-1.42) |  |
|  | **61-65** | 22,502 | 13,378 (59.5) | 1.13 (1.08-1.18) | 794 | 441 (55.5) | 1.39 (1.11-1.75) | 1,050 | 336 (32.0) | 1.14 (0.89-1.47) |  |
|  | **66-70** | 22,325 | 13,788 (61.8) | 0.89 (0.85-0.93) | 847 | 491 (58.0) | 1.10 (0.89-1.35) | 706 | 241 (34.1) | 0.94 (0.72-1.22) |  |
|  | **>70** | 18,039 | 10,005 (55.5) | Reference | 803 | 453 (56.4) | Reference | 455 | 139 (30.5) | Reference |  |
| **Education** | **Primary school** | 23,047 | 9,933 (43.1) | 1.93 (1.83-2.05) | 746 | 327 (43.8) | 1.47 (1.16-1.86) | 1,747 | 486 (27.8) | 1.42 (1.14-1.77) | 0.005 |
|  | **High school** | 50,389 | 29,139 (57.8) | 1.26 (1.20-1.33) | 1,322 | 699 (52.9) | 1.15 (0.94-1.41) | 1,792 | 628 (35.0) | 1.07 (0.87-1.32) |  |
|  | **1-4 years university** | 23,541 | 15,544 (66.0) | 0.98 (0.93-1.04) | 1,381 | 829 (60.0) | 0.97 (0.79-1.18) | 1,183 | 458 (38.7) | 1.00 (0.80-1.24) |  |
|  | **>4 years university** | 9,163 | 6,126 (66.9) | Reference | 642 | 379 (59.0) | Reference | 532 | 209 (39.3) | Reference |  |
| **Occupation** | **Retired** | 41,386 | 21,542 (52.1) | 1.20 (1.16-1.24) | 1,587 | 792 (49.9) | 1.55 (0.74-3.24) | 2,980 | 824 (27.7) | 1.87 (1.26-2.78) | 0.442 |
|  | **Unemployed** | 567 | 251 (44.3) | 1.53 (1.28-1.82) | 34 | 13 (38.2) | 1.31 (1.11-1.55) | 172 | 34 (19.8) | 1.30 (1.14-1.48) |  |
|  | **Employed** | 64,704 | 39,126 (60.5) | Reference | 2,597 | 1,453 (55.9) | Reference | 2,862 | 1,048 (36.6) | Reference |  |
| **Household**  **income (NOK)** | **≤484 000** | 25,500 | 10,715 (42.0) | 1.75 (1.66-1.84) | 1,076 | 429 (39.9) | 1.77 (1.41-2.24) | 2,639 | 661 (25.0) | 1.36 (1.11-1.66) | 0.134 |
|  | **484 001-755 000** | 26,776 | 15,393 (57.5) | 1.21 (1.16-1.26) | 955 | 525 (55.0) | 1.16 (0.95-1.41) | 1,480 | 511 (34.5) | 1.04 (0.85-1.27) |  |
|  | **755 001-1 130 000** | 27,083 | 16,791 (62.0) | 1.09 (1.05-1.13) | 971 | 580 (59.7) | 0.95 (0.79-1.14) | 1,186 | 458 (38.6) | 0.93 (0.76-1.13) |  |
|  | **>1 130 000** | 27,280 | 18,019 (66.1) | Reference | 1,216 | 724 (59.5) | Reference | 707 | 276 (39.0) | Reference |  |
| **Marital status** | **Single/widow** | 27,234 | 12,219 (44.9) | 1.39 (1.34-1.44) | 1,016 | 460 (45.3) | 1.16 (0.97-1.38) | 1,343 | 364 (27.1) | 1.21 (1.04-1.41) | 0.281 |
|  | **Cohabit/married** | 79,452 | 48,702 (61.3) | Reference | 3,199 | 1,796 (56.1) | Reference | 4,652 | 1,540 (33.1) | Reference |  |
| **Driving distance**  **(minutes)** | **> 40** | 21,078 | 11,132 (52.8) | 1.11 (1.07-1.14) | 635 | 300 (47.2) | 1.19 (1.01-1.41) | 733 | 207 (28.2) | 1.14 (0.99-1.32) | 0.406 |
|  | **21-40** | 39,610 | 22,101 (55.8) | 1.19 (1.15-1.23) | 1,203 | 623 (51.8) | 1.38 (1.13-1.69) | 2,161 | 676 (31.3) | 1.36 (1.12-1.65) |  |
|  | **≤ 20** | 44,285 | 27,064 (61.1) | Reference | 2,181 | 1,265 (58.0) | Reference | 2,813 | 987 (35.1) | Reference |  |
| **Use of antidiabetics** | **Yes** | 6,847 | 3,168 (46.3) | 1.38 (1.31-1.45) | 242 | 109 (45.0) | 1.27 (0.96-1.67) | 783 | 224 (28.6) | 1.06 (0.92-1.21) | 0.159 |
|  | **No** | 99,848 | 57,756 (57.8) | Reference | 3,979 | 2,149 (54.0) | Reference | 5,238 | 1,683 (32.1) | Reference |  |
| **Use of antipsychotics**  **and/or anxiolytics** | **Yes** | 8,740 | 3,461 (39.6) | 1.62 (1.54-1.70) | 240 | 98 (40.8) | 1.50 (1.13-1.99) | 329 | 89 (27.1) | 1.13 (0.95-1.34) | 0.004 |
|  | **No** | 97,955 | 57,463 (58.7) | Reference | 3,981 | 2,160 (54.3) | Reference | 5,692 | 1,818 (31.9) | Reference |  |

Odds ratios (ORs) for non-participation with 95% confidence interval (CI) deriving from multivariable logistic regressions. P-value for heterogeneity was calculated to evaluate differences in ORs between individuals born in Norway, individuals born in Western countries and individuals born in non-Western countries.

**
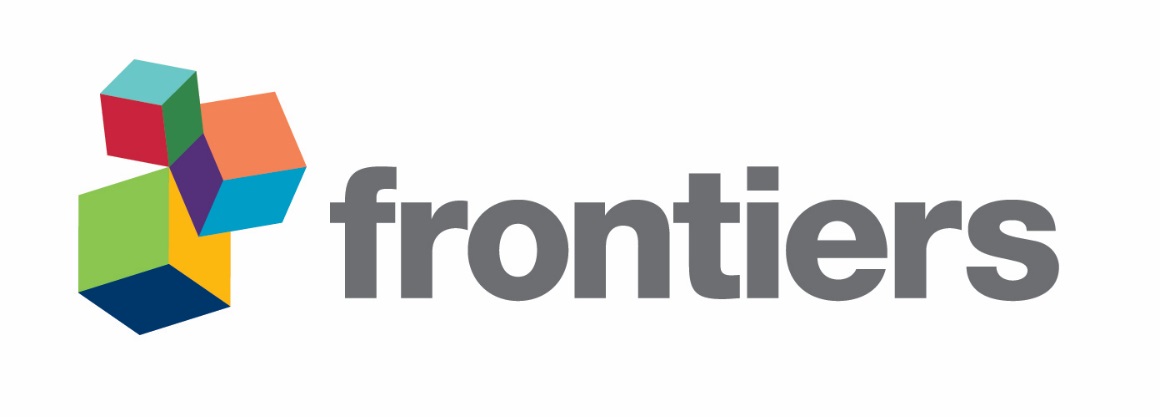
**
